# Mixing injector enables time-resolved crystallography with high hit rate at X-ray free electron lasers

**DOI:** 10.1063/1.4961971

**Published:** 2016-08-29

**Authors:** George D. Calvey, Andrea M. Katz, Chris B. Schaffer, Lois Pollack

**Affiliations:** 1 School of Applied and Engineering Physics, Cornell University, Ithaca, New York 14853, USA; 2 Meinig School of Biomedical Engineering, Cornell University, Ithaca, New York 14853, USA

## Abstract

Knowledge of protein structure provides essential insight into function, enhancing our understanding of diseases and enabling new treatment development. X-ray crystallography has been used to solve the structures of more than 100 000 proteins; however, the vast majority represent long-lived states that do not capture the functional motions of these molecular machines. Reactions triggered by the addition of a ligand can be the most challenging to detect with crystallography because of the difficulty of synchronizing reactions to create detectable quantities of transient states. The development of X-ray free electron lasers (XFELs) and serial femtosecond crystallography (SFX) enables new approaches for solving protein structures following the rapid diffusion of ligands into micron sized protein crystals. Conformational changes occurring on millisecond timescales can be detected and time-resolved. Here, we describe a new XFEL injector which incorporates a microfluidic mixer to rapidly combine reactant and sample milliseconds before the sample reaches the X-ray beam. The mixing injector consists of bonded, concentric glass capillaries. The fabrication process, employing custom laser cut centering spacers and UV curable epoxy, ensures precise alignment of capillaries for repeatable, centered sample flow and dependable mixing. Crystal delivery capillaries are 50 or 75 *μ*m in diameter and can contain an integrated filter depending on the demands of the experiment. Reaction times can be varied from submillisecond to several hundred milliseconds. The injector features rapid and uniform mixing, low sample dilution, and high hit rates. It is fully compatible with existing SFX beamlines.

## I. INTRODUCTION

Proteins are central to all aspects of cellular life, from metabolism to facilitated diffusion across membranes, to defense against foreign particles. Techniques, such as protein crystallography^1^ and nuclear magnetic resonance spectroscopy,^2^ have been successfully applied to solve protein structures, providing critical insight into how these remarkable molecules carry out their diverse functions. However, proteins are dynamic molecules, and a complete understanding of how they work requires knowledge of functional, structural intermediates.^3^ Because states that are critically linked to enzymatic function can be populated on time scales of microseconds to milliseconds,^4,5^ the development of new techniques to measure high resolution structural information on these timescales is essential.

Time-resolved crystallography aims to solve structures of intermediate states by initiating a reaction within a protein crystal, then obtaining X-ray diffraction patterns from the crystal a short time later. Most prior work focused on light-activated processes in proteins: the crystal is “pumped” with a pulse of laser light to initiate a structural change, and the altered state is then “probed” by the X-ray beam.^3,6^ However, in cells, reactions are more commonly induced by chemicals, where a substrate or activator molecule initiates a conformational change in a protein. Some reactions of this type have been studied with time-resolved crystallography,^7^ but the relatively large crystals required for synchrotron crystallography (hundreds of microns) introduce complications. Reactant molecules diffuse slowly into such a large crystal. Half-saturation binding with small ligands can take minutes or longer,^7,8^ precluding observation of many faster reactions of interest.

The recent development of high intensity X-ray free electron lasers (XFELs) and Serial Femtosecond Crystallography (SFX) allows high resolution structure determination from small crystals ranging in size from about 200 nm to several μm.^9,10^ In an XFEL experiment, each extremely intense X-ray pulse destroys solid samples. Many sample delivery techniques have been developed to circumvent this problem, each with unique benefits.^11–14^ Most frequently, crystals are delivered to the X-ray beam in the form of a free liquid jet produced by an injector such as a Gas Dynamic Virtual Nozzle (GDVN).^15^ In a GDVN, liquid jetting from a central capillary is focused down through a small orifice by a high pressure gas sheath flow,^11,16,17^ producing a micron scale liquid jet that is stable at flow rates as low as several *μ*l/min.^11^ The high speed of the GDVN jet ensures that the sample, micron scale crystals in solution, is replenished between each pulse. The success of this technique extends beyond static crystallography to light-activated, time-resolved reactions.^18,19^ The successful observation of such small crystals gives hope to the possibility of fast chemically activated time-resolved crystallography.^20^

The success of a mix-and-inject experiment depends on the development of new injectors that rapidly and efficiently mix reactant with crystals milliseconds before delivering the reacting species into the X-ray beam. A proof-of-principle mixing injector, developed by Wang *et al*, was a variation of the GDVN with the central sample line replaced by coaxial supply lines for crystal solution and reactant solution.^21^ Within this device, the two solutions mixed by diffusion prior to focusing into a free liquid jet. While representing an important first step toward the development of a mixing injector for SFX, several aspects of this injector's operation were not yet optimized. To achieve fast mixing, the device needs to operate at a high reactant-to-crystal solution flow rate ratio (high crystal dilution), ∼2000:1, which significantly reduces the percentage of X-ray pulses that hit a crystal (the “hit rate”). The low hit rate may prevent the collection of sufficient diffraction patterns to solve a structure over the course of a normal XFEL experiment. This device has a nominal submillisecond mixing time for some reactants; however, this calculation only considers the diffusion time after hydrodynamic focusing and neglects time the protein is in contact with the substrate before full focusing occurs, which could be much longer than the mixing time due to low sample velocity and slow focusing. This effect, known as premixing, lengthens the uncertainty in mixing times.^22,23^ Finally, the high fluidic resistance of the 20 *μ*m inner diameter line requires high driving pressures and may be prone to clogging when long supply lines are present.^24^

Here, we present a new mixing injector that improves upon previous work while retaining the beneficial characteristics of the GDVN. It features rapid mixing with orders of magnitude less dilution than the previous design, resulting in a much higher hit rate. The premixing time for realistic flow conditions can be well below 1 ms. The crystal supply line can be made with 50 or 75 *μ*m inner diameter tubing, which allows this injector to operate at lower pressures and reduces its risk of clogging. Additionally, this injector is compatible with existing sample environments at the CXI beamline (LCLS) and with the DAPHNIS platform at SACLA,^25^ making it usable at both XFEL beamlines commonly used for SFX. This device is therefore ideal for time-resolved mix-and-inject crystallography experiments at XFELs. Both detailed fabrication and characterization protocols are described below.

### A. Description of mixing injector

**FIG. 1.**
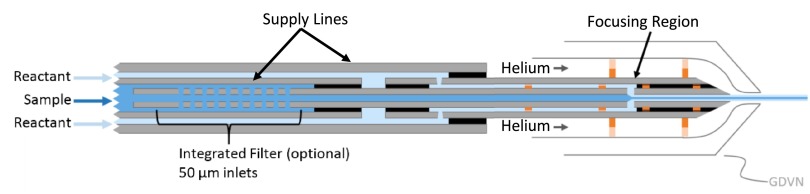
Schematic of the mixing injector. Black areas indicate regions bound by UV epoxy. Polyimide centering spacers are shown in orange. The inner sample line in the GDVN can be either 50 or 75 *μ*m ID. The length of the device from the tip of the GDVN to the supply lines is approximately 10 cm.

A schematic of the mixing injector is shown in Figure 1. The device is composed of concentric glass capillaries. Crystal-containing solution flows through the inner capillary, while the reactant solution flows through the outer capillary. In the focusing region, the crystal solution is accelerated and hydrodynamically focused into a micron-sized thin jet. Reactant molecules from the outer stream rapidly diffuse into and across the thin inner jet, initiating a reaction in the proteins within the crystals. The reaction progresses as the crystals travel from the focusing region to the end of the mixing injector. The device terminates in a standard GDVN nozzle, where the mixed solution is injected into the X-ray chamber as a free liquid jet.

The mixing injector is a versatile tool which is easily adapted to accommodate different experimental requirements and sample environments. It can be fabricated with or without an integrated filter positioned just upstream of the mixing region. Only fused silica, polyimide, and UV curable epoxy come into contact with the sample or reactants. These materials have exceptional chemical compatibility and can transport solutions with widely varying pH and salt concentration. For experiments at CXI/LCLS, mixing injectors have long supply lines that traverse the nozzle rod, allowing transport of sample from external reservoirs to the mixer, which resides inside the vacuum chamber. For experiments in the helium environment of SACLA's DAPHNIS platform, the design is simplified because long supply lines are not required.

## II. DEVICE FABRICATION

**FIG. 2.**
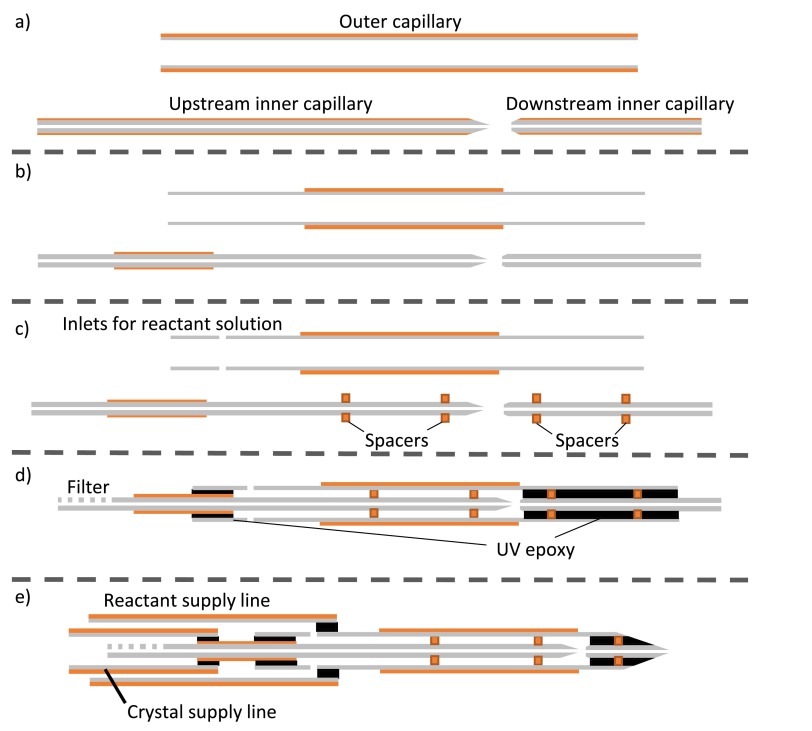
Overview of the mixer fabrication process. (a) Capillaries are cut to length and polished to flatten and bevel their ends. (b) Polyimide coating is removed from select portions of the capillaries. (c) Polyimide centering spacers are placed around the inner capillary, and reactant inlet holes added to the outer capillary. (d) The inner capillaries are inserted into the outer and bonded in place with UV curable epoxy, indicated in black. If desired, a filter is laser cut into the upstream inner capillary prior to bonding. (e) The mixer tip is polished to a point and supply lines are added.

An overview of the mixer fabrication process is shown in Figure 2.

### A. Step 1: Capillary preparation and polishing

Glass capillaries with standard polyimide coating (Polymicro Technologies and Postnova Analytics Inc.) comprise the mixing injector. For each mixer, we use a diamond tipped scribe to cut a 75 mm long and a 180 mm long piece of small capillary (50 or 75 *μ*m ID and 193 *μ*m OD). These pieces will form the inner crystal line. We cut a 150 mm long piece of a larger capillary (280 *μ*m ID and 370 *μ*m OD) for the outer reactant line.

**FIG. 3.**
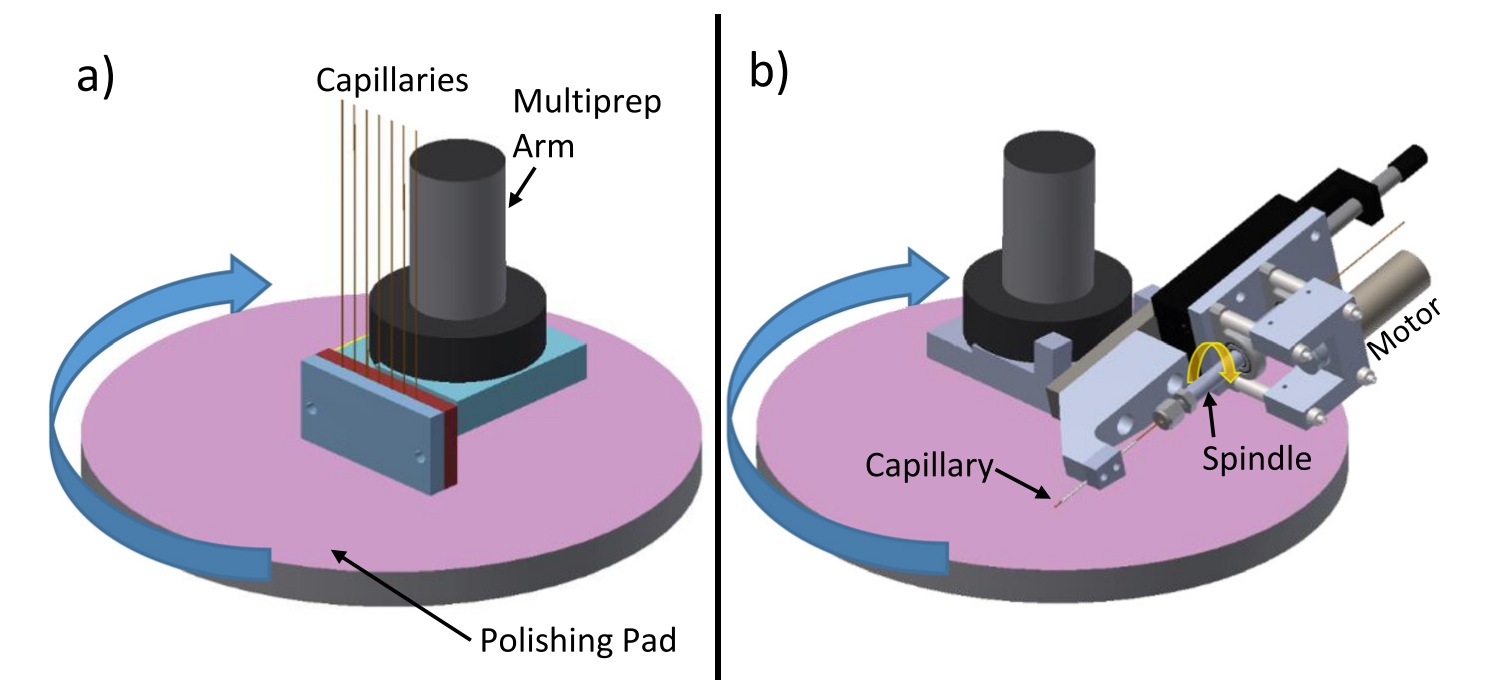
Schematics of capillary polishing procedures. (a) Custom chuck for flattening the ends on capillaries. (b) Custom jig for beveling the end of capillaries.

The uneven, jagged edges of these scribed capillaries must be removed to avoid fabricating fragile devices with undesirable flow characteristics. First, we polish flat the cleaved edges of all capillaries with a custom chuck fabricated for an Allied MultiPrep polishing system (Allied High Tech Products, Inc.). The chuck, shown in Figure 3(a) holds the capillaries perpendicular to the polishing pad. It is lowered into the pad to polish off jagged edges and flatten the capillary. We initially polish off ∼300 *μ*m of capillary at a rate of 2 *μ*m/s using a 15 *μ*m grit polishing pad. This procedure creates a flat capillary end but leaves the glass with a rough texture. Polishing an additional 50 *μ*m into the capillary with a 3 *μ*m grit polishing pad leaves a smooth, flat end.

Additionally, the edges of the capillaries comprising the inner crystal line are beveled to facilitate insertion of the capillary into the slightly smaller centering spacers in a later step. To bevel the edges, we use the custom jig shown in Figure 3(b) to hold the capillary at a 10° angle to the polishing pad. A motor on the jig constantly spins the capillary in the opposite direction of travel to the polishing pad. The spinning capillary is lowered onto the 3* μ*m grit polishing pad to bevel the edges. This grit removes material quickly while producing a bevel with low roughness. A schematic of the capillaries at this point in the fabrication process is shown in Figure 2(a).

### B. Step 2: Coating removal

The capillaries are produced with a polyimide coating which adds strength but blocks UV light. Therefore, the coating must be removed from the parts of the capillaries that will later be bonded with UV curable epoxy. Additionally, removal of the coating from the inner capillaries provides more clearance for centering spacers and reactant solution. The downstream inner capillaries are completely stripped by heating at 600 °C for one hour. We remove the coating from the ends of the polished upstream inner and outer capillaries by immersing in sulfuric acid heated to about 110 °C. The remaining coating is left intact and adds strength to the completed device. Figure 2(b) shows the capillaries after coating removal.

### C. Step 3: Laser cutting the centering spacers and reactant inlet holes

**FIG. 4.**
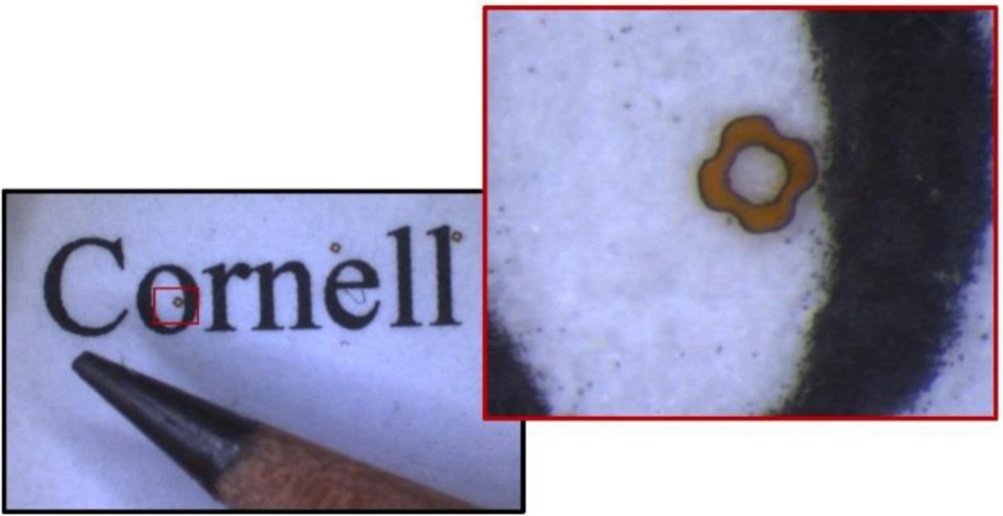
Laser cut spacer shown with a pencil tip and size 10 font for scale. The shape of the spacer is designed to allow liquid to flow past in the assembled device.

To hold the inner capillaries aligned and concentric, the spacers are sized to grip tightly on the inner capillaries and fit snugly inside the outer capillaries. The shape of the spacers allows reactant fluid to flow past. We use femtosecond laser ablation (setup described in Ref. 26) to cut spacers from a sheet of 2 mil polyimide. To prevent movement during ablation, a thin layer of glycerol laminates the sheet onto a plate of aluminum. An Olympus 4×, 0.28 NA objective focuses the laser, and a Newport three-axis stage moves the polyimide through the focus at 170 *μ*m/s along a trajectory to cut the outline of the spacers. Pulses with a central wavelength of ∼800 nm, a pulse duration of ∼50 fs, and 3.5* μ*J per pulse energy (measured at the sample) are delivered continuously at 1 kHz. This energy is a compromise between processing time and cutting fidelity. Higher power can cause charring or movement of the polyimide during cutting. Each spacer is cut in about 30 s. After cutting is complete, the sheet of polyimide is pulled off the aluminum, leaving the majority of the spacers fixed on the metal. Figure 4 shows a completed spacer.

Two 50* μ*m inlets, positioned on opposite sides of the outer capillary, allow reactant solution to flow in from the supply line and are cut with the same laser setup, but at a slightly lower speed of 100 *μ*m/s. The focus of the laser is first positioned inside the open area in the capillary and then moved outward in a helical-like path to cut out a small cylinder of glass (trepan-drill) which is blown away by compressed air. The laser power was set to ∼3.5* μ*J per pulse, and each hole is cut in approximately one minute.

If the device is fabricated with an integrated filter for the protein crystal solution, 10 additional inlets on the inner capillary are cut in a similar manner after inserting the inner capillary into the outer. The laser is focused into the center of the capillary then moved along a path to completely ablate a cylindrical hole approximately the size of the capillary ID.

### D. Step 4: Spacer positioning

**FIG. 5.**
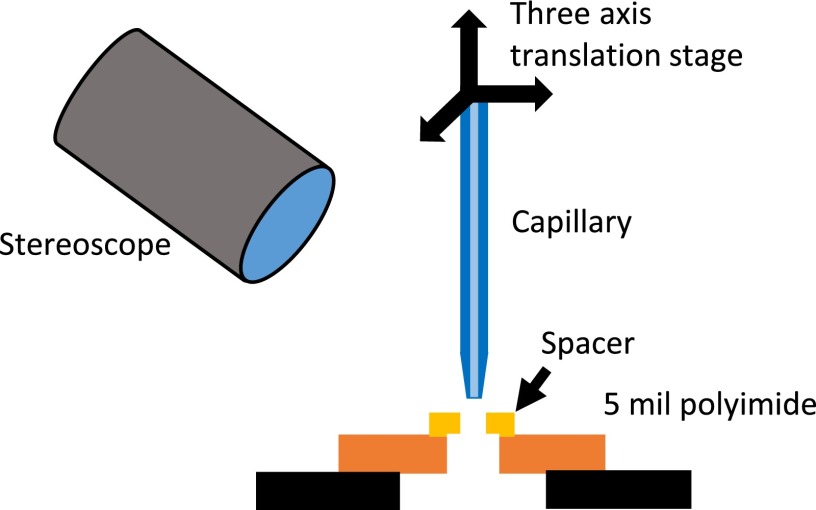
Spacer positioning setup. A three-axis translation stage and a stereoscope are used to position the beveled capillary over the hole in a centering spacer. The capillary is pushed through the spacer into the gap beneath the polyimide sheet.

To place the spacers around the inner capillary, we use the positioning setup illustrated in Figure 5. A sheet of 5 mil polyimide with a laser cut hole that is larger than the spacer's ID but smaller than its OD is stretched tightly over a metal aperture. A spacer is then placed on top of the sheet with its hole concentric. The spacer cannot fall through or be pushed through the hole in the sheet due to the larger OD of the spacer. We align the beveled end of the capillary over the hole in the spacer and spear the capillary through. The spacer must expand slightly to accommodate the larger ID of the capillary, resulting in an extremely tight fit. We continue to push the capillary further through the spacer until the spacer is ∼6 mm from the tip of the capillary. We then add a second spacer just past the capillary tip. Figure 2(c) shows the capillaries after the addition of the spacers.

### E. Step 5: Mixer assembly

We use a micrometer driven stage to adjust the spacing between the upstream and downstream inner capillaries in the assembled and unbonded device to ∼50 *μ*m. While observing the device with a microscope, we place a small drop of UV curable epoxy (UV15, Masterbond) near the end of the outer capillary. The epoxy wicks into the gap between the inner and outer capillaries and flows around the spacers. When it reaches the edge of the desired bond region, we rapidly cure it with light from an HBO lamp in a Zeiss Axiovert 130 microscope (Carl Zeiss AG). The process is repeated for the other end. The bonded capillaries are shown in Figure 2(d).

After curing, we scribe the downstream end of the bonded mixer to length with the femtosecond ablation laser. This procedure is necessary as mechanical scribing methods fail to cut through the epoxy without crushing the capillaries. To facilitate jetting, we bevel the end of the mixer to a point using the MultiPrep polisher with the same beveling jig as in step 1. Initially, we use a 15 *μ*m grit polishing pad to remove material rapidly, then a 3 *μ*m grit pad for a smooth finish. For mixers to be used at CXI, we bond long supply lines for both the crystal and reactant solutions to the completed mixer using the same procedure as described above. A schematic of a finished mixer with supply lines is shown in Figure 2(e).

### F. Step 6: Building the mixing injector

**FIG. 6.**
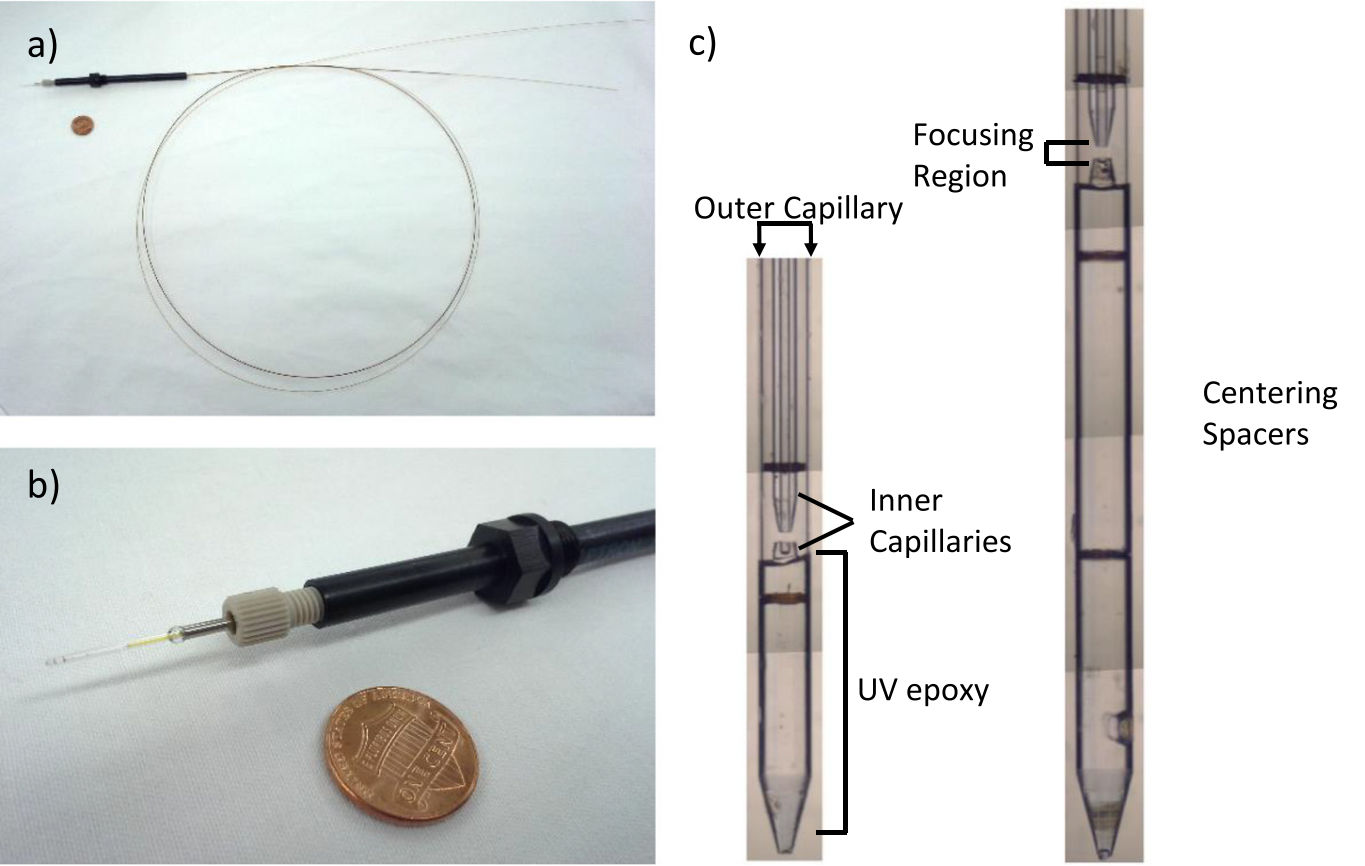
(a) Picture of a completed mixing injector with 1.2 m long supply lines. The black PEEK part is the nozzle holder described in Refs. 15 and 27 with an additional protective tube to cover bonded regions. A penny is included for scale. (b) Close up picture of the nozzle holder and tip of the injector. The outer capillary of the mixer is barely visible inside the glass shroud of the GDVN. (c) Optical composite images of two completed mixers before injector assembly. The different delay lengths (approximately 2 mm and 4 mm) after the focusing region allow different reaction times to be probed. Note the hypodermic needle-like tip of the longer mixer. This results from a slightly off-centered tip of the inner capillary in this region since the centering spacers are several mm away.

We fabricate the glass shroud of the GDVN according to standard protocols for SFX experiments.^11,27^ Briefly, we flame polish the ends of.75 mm ID, 1.0 mm OD glass tubes (Sutter Instruments) to produce a narrow aperture, then add a taper to the end of the tube with the MultiPrep. We complete the GDVN shroud by gluing the polished glass tube inside of a short piece of 1/16 in. steel tubing. Polyimide spacers position the mixer in the center of the glass shroud. A nozzle holder, described in Refs. 15 and 27, grips the mixer and shroud and provides concentric gas flow. The completed device is shown in Figure 6.

## III. DEVICE CHARACTERIZATION

**FIG. 7.**
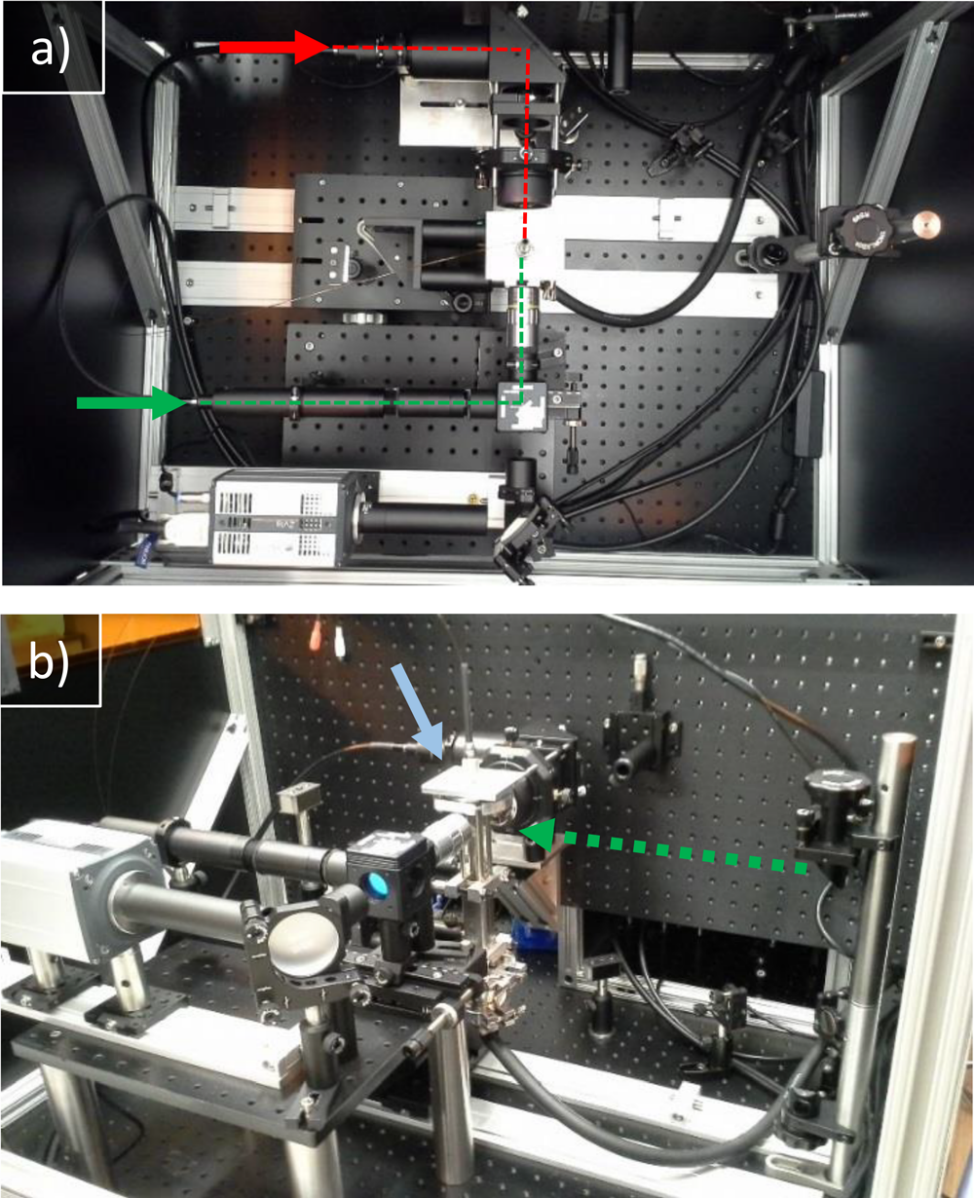
(a) Top down image of the microscope. Arrows indicate liquid light guide attachments. Red lines indicate the light path for backlight, and green lines indicate the light path for epi-illumination. (b) Side image of the same setup. The dashed green arrow shows the light path of the pulsed laser. The blue arrow indicates the vacuum chamber.

We characterized the mixing injectors using a custom-built long working distance fluorescence microscope. The microscope features two options for fluorescence excitation: epi-illumination from a XPE2 green LED (Cree, Inc.) and side illumination from a Sol 532 3 W pulsed DPSS laser (Bright Solutions). An additional Cree XPE2 red LED provides Kohler backlight for brightfield imaging. We collected images with a Zyla 5.5 camera (Andor Technology) and a 10× infinity corrected long working distance objective. (Mitutoyo). Inside the microscope, the mixer was mounted to a custom windowed vacuum chamber, a gift from Arizona State University, to image the liquid jet in vacuum. The microscope is shown in Figure 7.

When fabricated according to the protocols discussed above, our mixing injectors are robust and function over a broad range of pressures and flow rates. Mixers with integrated filters typically require less than 4000 mbar to drive a sample solution at a flow rate of several *μ*l/min due to low back pressure from the larger supply capillary. CXI-compatible mixers with no integrated filter attached do not step up to a larger diameter supply line to traverse the nozzle rod and therefore have higher back pressure in the crystal capillary. Flow through the outer capillary can be driven up to a few hundred *μ*l/min with less than 8000 mbar of pressure. The exact pressures required for both lines depend on the geometry of the gas nozzle and the pressure of the helium gas at the tip. All mixing injectors characterized here were equipped with an integrated filter and large crystal supply line, allowing us to control the liquid flow for both inner and outer lines with an OB1 pressure controller and MFS flow meters (Elveflow). In the case of viscous solutions or mixers without integrated filters, solutions can be driven at higher pressures without damaging the device, using an HPLC pump or high pressure gas.

**FIG. 8.**
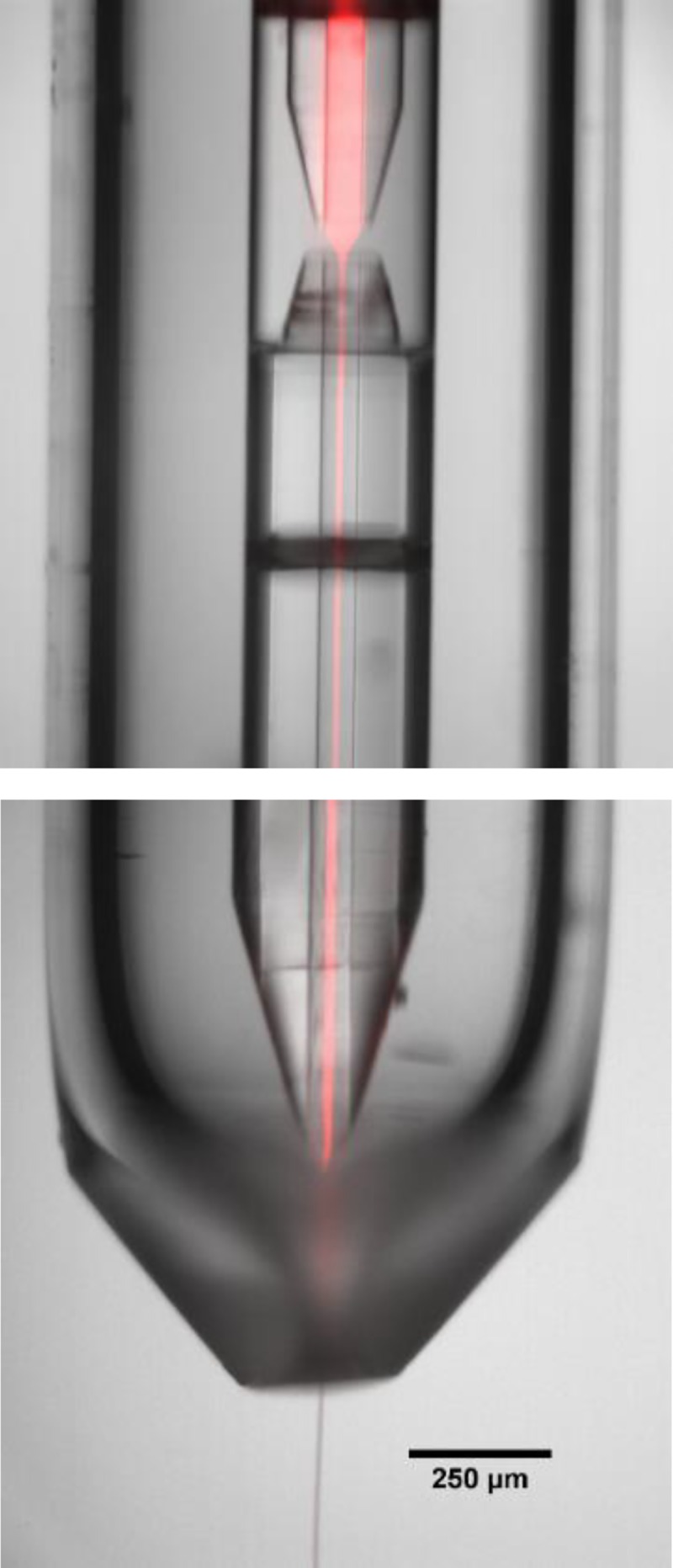
Composite image of fluorescent dye flowing in the inner channel of the mixer, while the injector jets into vaccum. The red color in the image is a false-color overlay of a fluorescence image onto a background brightfield image of the mixing injector.

To visualize the liquid flow through the device, we pumped 10 *μ*M Rhodamine 6 G dye through the central capillary and water through the outer capillary. Figures 8 shows the dye hydrodynamically focused to a thin, centered jet after the focusing region. This thin jet is essential for fast diffusion of reactant into a crystal solution. Protein crystals flowing through the mixer follow the same flow path.

**FIG. 9.**
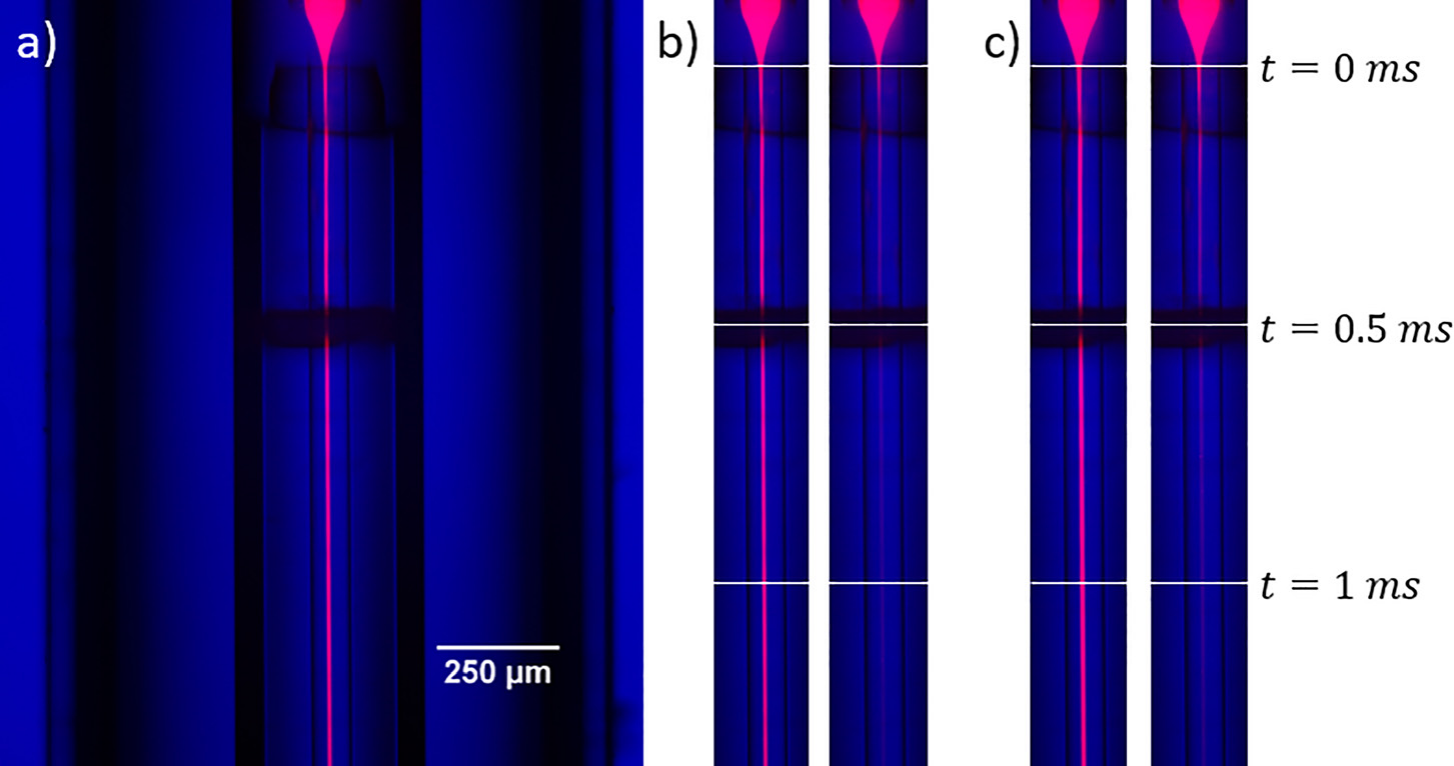
(a) Composite image showing rhodamine dye (red) superimposed on a brightfield image of the mixing injector (blue). Here, water is flowing through the outer capillary. The dark band midway through the image is a centering spacer. (b) Composite images of the mixer's central channel with outer:inner flow rate ratio of 40:1 for rhodamine dye in the central capillary and water (left) or iodide (right) in the outer capillary. The total flow rate is ∼62 *μ*l/min. White horizontal lines indicate time elapsed along the central jet. (c) Same as in (b), but with outer:inner flow rate ratio of 30:1.

To characterize mixing within this injector, we flowed 20 μM rhodamine dye through the inner capillary and 300 mM potassium iodide in the outer capillary. Iodide provides a non-radiative pathway for excited fluorophores, decreasing/quenching the fluorescence from the rhodamine.^22,28^ Figure 9 shows fluorescence images of both quenched and unquenched rhodamine at iodide:rhodamine flow rate ratios of 40:1 and 30:1. In both cases, the fluorescence decreased by half after ∼0.25 ms, and nearly reached its minimum level after 1 ms. Importantly, most mixing experiments are designed with the reaction threshold well below 100% of the full reactant concentration in the outer capillary. Therefore, this quenching experiment demonstrated that this device can comfortably achieve submillisecond mixing times.

Another important aspect of the mixing injector is the characteristics of the free jet it produces. Typical devices produced a stable jet for total flow rates above 10 to 20 *μ*l/min, performance comparable to non-mixing GDVNs (∼10 *μ*l/min).^29^ In a mixing experiment, devices are designed so that the total flow rates will exceed this number (see discussion), so jet instability is not a concern.

**FIG. 10.**
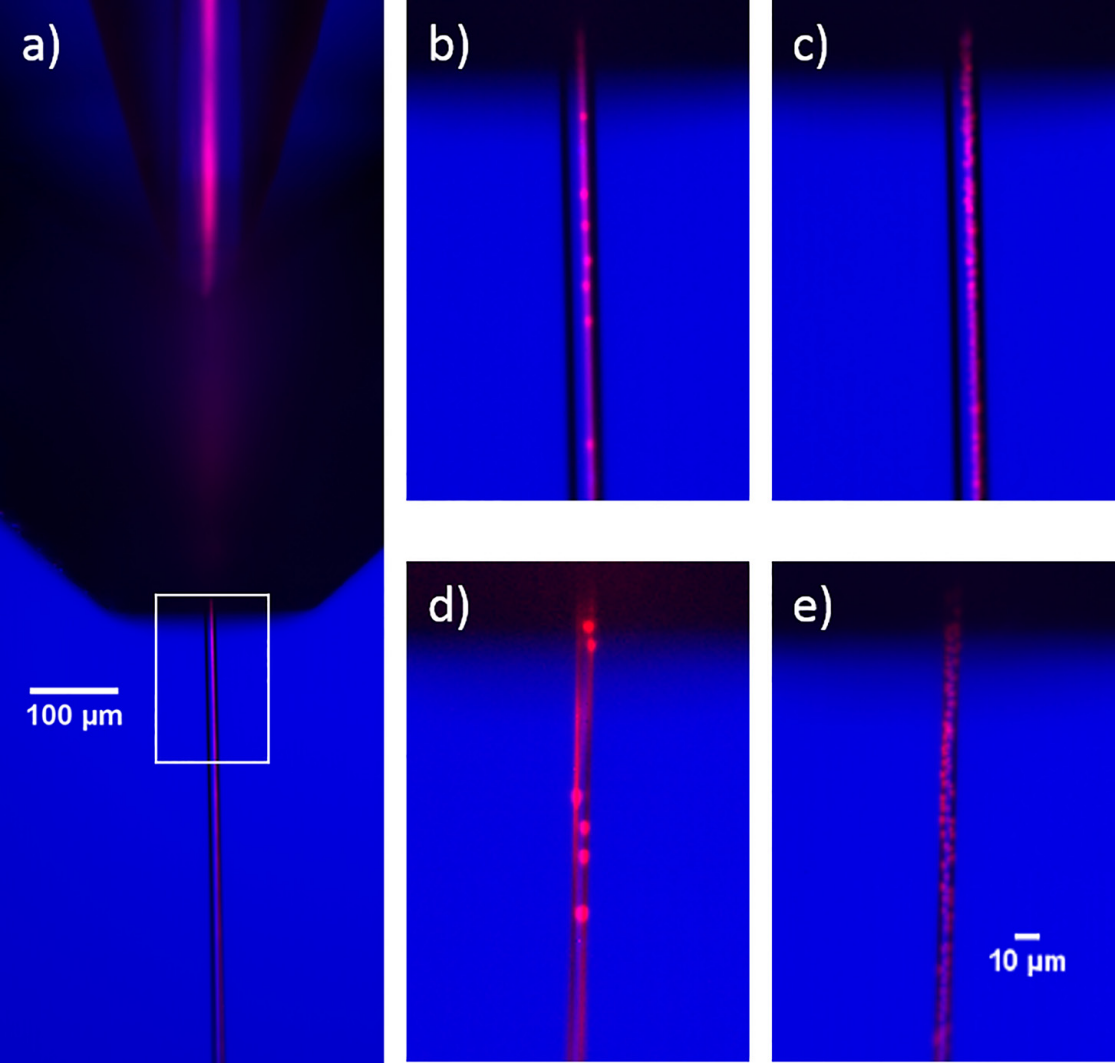
Composite images of fluorescent beads and dye (red) overlaid with brightfield images (blue). Fluorescence was excited with the Sol laser pulsing at 10 kHz and approximately 200 *μ*J per pulse. 40 fluorescence images per condition were acquired, each with an exposure time of 9 *μ*s. (a) Image showing co-focused beads and dye inside the mixer and in the free jet. The white box represents the region of interest expanded in the other images. (b) Representative image (from set of 40) showing focused dye and beads in the free jet with flow rates of 120 and 3 *μ*l/min in the outer and inner line. (c) Max intensity projection of all 40 fluorescent images from the set described in (b). (d) Representative image with 30 and 3 *μ*l/min outer and inner flow rates. The focused dye spirals one complete revolution around the outside of the jet. The beads show some preference to follow the dye, but are partially scrambled. (e) Max intensity projection of all 40 fluorescent images from the set described in (d).

We briefly investigated the flow focusing characteristics inside of the free jet. Flow focusing, where crystals flow in a smaller stream within the free jet, is desirable since the hit rate will be higher than if the crystals were fully dispersed. To study the flow focusing capabilities of the device, we flowed 2 *μ*m red fluorescent polystyrene beads (Sigma Aldrich) along with 20 *μ*M rhodamine in the central capillary and water in the outer capillary. The beads and dye are always co-focused inside of the device. Because the tip of the mixer is beveled asymmetrically like a hypodermic needle, we did not observe any recirculation cells in the meniscus.^30^ After the gas aperture, the dye always remained focused in the free jet. However, as shown in Figure 10, the beads sometimes remained co-focused with the dye and sometimes scrambled throughout the jet. The tendency towards one mode or the other seemed to depend on several factors, including the flow rate, nozzle geometry, particle size and, potentially, shape. Further characterization is needed to determine if we can leverage flow focusing for time-resolved SFX experiments.

## IV. DISCUSSION

### A. Logistics of time-resolved SFX

The goals of a time-resolved crystallography experiment are to observe discrete intermediate structures that occur as the protein functions and to determine the lifetime of these states. The diffraction pattern obtained from a reacting crystal will give the average electron density of the proteins. Although this average density can vary continuously as a function of time, the electron density of an individual protein progressing through a reaction moves quickly before pausing in longer-lived intermediate states that represent local minima in the free energy landscape. Singular value decomposition can extract intermediate states from the average density; however, it is essential to have enough proteins in the same intermediate state at the same time to produce a detectable signal from that state.^31^

**FIG. 11.**
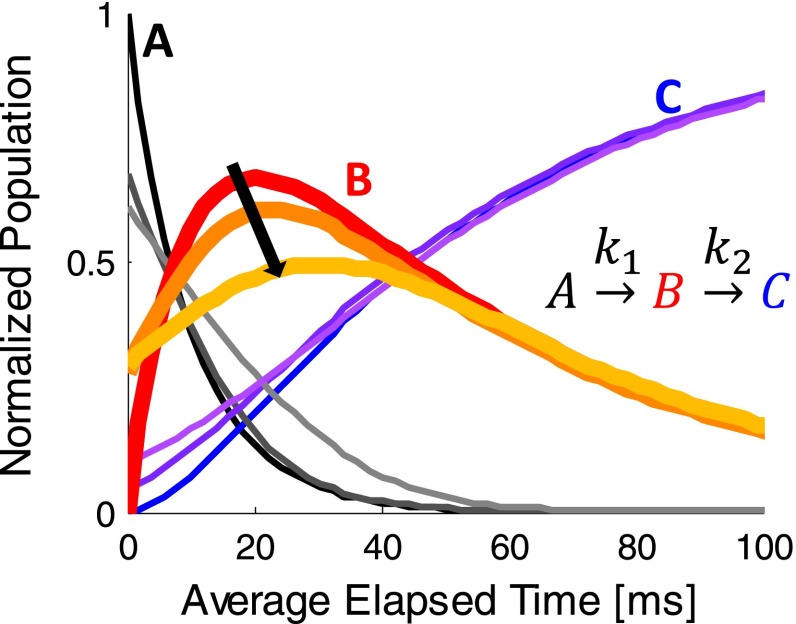
Theoretical concentrations for all three states in a two-step reaction. As timing dispersion increases, the trend is towards lighter colors. For state B, the curves progress in the direction of the arrow as dispersion increases from 0 to 10 ms to 20 ms.

The observed occupancy of a given intermediate state is maximized when there is minimal dispersion (or variation) in the elapsed time between reaction initiation and X-ray diffraction for each subunit in the crystal. Figure 11 shows a simple example of a two-step reaction where species B is the transient state to be observed. The reaction is plotted for three different amounts of dispersion: 0, 10, and 20 ms. The red curve, representing zero dispersion, maximizes the peak concentration of protein in state B and has the sharpest features. Probing state B at the time corresponding to its peak concentration gives the best possible signal. As timing dispersion increases, the peak concentration shifts down and to the right, represented by the orange and yellow curves. This effect decreases the maximum signal and, therefore, the possibility of observing state B. Clearly, it is important to keep timing dispersion at an acceptable level.

Ideally, each protein in the crystal reaches the desired reactant concentration at the same time, and the subsequent reaction proceeds for the same amount of time before encountering the X-ray beam. In reality, timing dispersion is unavoidable. Both diffusion into the crystals and mixing in the device cause a distribution in the time at which subunits are exposed to reactants. Neither of these effects can be completely eliminated, and there are trade-offs of signal strength vs. fast diffusion into crystals, and hit rate vs. fast mixing in the device that must be carefully balanced for each experiment.

#### 1. Diffusion into crystals

Perhaps, the largest barrier to reaching the ideal case of instantaneous mixing arises from diffusion into the crystals themselves. All transport inside protein crystals occurs along the solvent channels between the proteins. Although protein crystals can have large solvent contents by volume, typically around 50%,^32^ these channels form a torturous path for reactants to diffuse along. Consequently, the diffusion coefficient must be modified when considering a crystal as a homogeneous medium. Modeling the solvent channels as randomly oriented cylinders provides an accurate estimate of the effective diffusion coefficient, $D_{eff}$.^8^ In this case, $D_{eff}$ is related to the accessible volume Φ by^33^

$$D_{eff}=\Phi^{2}D.$$ (1)

For an example of this effect, consider glucose diffusing into crystals of glycogen phosphorylase b, a system with 26% accessible volume.^8^ Using $D_{eff}$, the calculated diffusion time for the reactant concentration to reach 69% in the center of a 1 × 2 × 3 *μ*m crystal is 2.2 ms, 15 times longer than the 0.15 ms predicted using the diffusion coefficient in solvent.^20^ This effect can be even more pronounced for other systems, such as fluorescein diffusing into lysozyme crystals (3% accessible volume). Here, diffusion occurs approximately 1000 times slower than fluorescein diffusing in bulk solvent.^8^ For systems similar to this, the slow diffusion would result in proteins on the outside of the crystal binding ligands and beginning to react significantly before those in the middle for all but the smallest crystals. This relationship determines the upper bound on crystal size for a given reaction. Careful consideration and characterization of this effect for a particular crystal/reactant system is critical when exploring the feasibility of time-resolved crystallography.

#### 2. Device mixing times

In addition to the dispersion in reaction initiation time from the diffusion into the crystals, the contribution from the mixer must also be carefully considered. The outer part of the sample stream is exposed to higher reactant concentration before the inner part of the sample stream, leading to variable reaction initiation time depending on the location of the crystals within the sample stream. Minimizing this effect not only sharpens the time resolution of the experiment but also, more importantly, it increases the signal strength by maximizing the population in intermediate states. However, low dispersion and fast mixing can come at the expense of hit rate, an effect that is discussed below.

For simplicity, mixing in this device can be broken down into two parts: premixing, occurring in the region between the two inner capillaries where the sample is being focused, and mixing, occurring in the downstream inner capillary after focusing.

### B. Premixing

Premixing refers to diffusion that occurs before the sample stream is fully focused.^22,23^ During focusing, the inner liquid is funneled into a smaller sample stream, accelerating as it thins. Sample on the outer edge of the stream begins reacting before sample in the middle of the stream. Minimizing this premixing effect is critical to short mixing times and to initiating a conformational change uniformly across the sample jet.

**FIG. 12.**
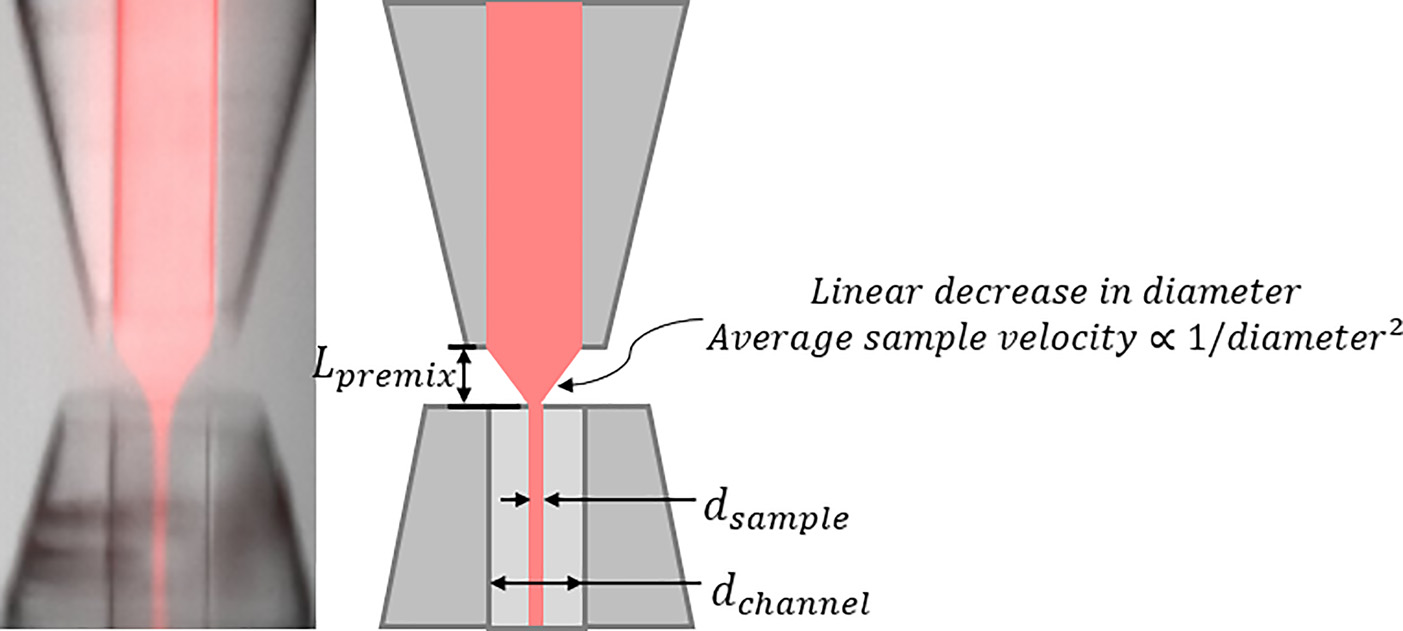
(Left) Detailed image of the focusing region. (Right) Schematic showing distances relevant to the discussion.

To estimate the effect of premixing in our device under typical flow conditions, we adapt the premixing time from Refs. 23 and 22 as the average time for the sample to traverse the focusing region. The only significant contribution to the premixing time is the time to traverse the gap from the upstream to downstream inner capillaries, shown as *L_premix_* in Figure 12. By approximating the flow as a linear decrease in diameter over this distance, the average time to traverse it can be estimated and is short, 0.5 ms for 5 *μ*l/min sample flow in a mixer with 50 *μ*m channels, or approximately 2 ms for 1 *μ*l/min. The additional time in the downstream capillary before full focusing is computed by dividing the entrance length, *L_e_*, by the velocity, *v*

$$\frac{L_{e}}{v}=0.05\times \frac{\rho d_{channel}^{2}}{\eta },$$ (2)

where *ρ* is the density of the liquid and η is the viscosity.^34^ In our design, this additional time is negligible, in the order of 100 *μ*s. The constriction created by the downstream capillary is a unique feature of our device that significantly reduces premixing. In a different device without this constriction, *L_premix_* and the premixing time could be orders of magnitude larger, making the constriction absolutely critical for millisecond time-points.

The average time to cross *L_premix_* in our device for a given flow condition is roughly an order of magnitude less than the experimental time-point that would be measured for that condition. Therefore, the contribution to timing dispersion from premixing is negligible in this design.

### C. Mixing times

Following the rapid focusing, reactants diffuse quickly across the thin sample stream. Since premixing can be neglected for this device, we define the mixing time as the time after focusing for the reactant concentration to reach a threshold level at the center of the sample stream. For a given device, the flow conditions determine the mixing time and must be chosen as a compromise between both time resolution and signal from transient states and the X-ray hit rate.

Mixing is characterized by the convection-diffusion equation

$$D\nabla^{2}c(r,t)-\nabla \cdot c(r,t)v(r,t)=\frac{\partial c(r,t)}{\partial t},$$ (3)

where c is the concentration of reactant in the sheath flow, t is time, D is the reactant's diffusion coefficient, ***r*** is the position, and ***v*** is the velocity. The mixer operates at steady state, the liquid is incompressible, and the velocity only has a component along the channel in a Poiseuille flow profile. Therefore, the equation simplifies to

$$D\left(\frac{\partial^{2}}{\partial r^{2}}+\frac{\partial^{2}}{\partial z^{2}}\right)c(r)=u_{\max}\left(1-\frac{4r^{2}}{{d_{channel}}^{2}}\right)\frac{\partial c(r)}{\partial x},$$ (4)

where $d_{channel}$ is defined in Figure 12. For this flow, the Peclet number, which characterizes the ratio of flow transport to diffusion along the channel, is in the order of 10^4^, meaning diffusion along the channel can be neglected and we need only consider diffusion in the radial direction.

We are interested in the solution near the center of the channel because the sample stream is small; therefore, we can further approximate by neglecting the $4r^{2}/{d_{channel}}^{2}$ term. This allows us to convert the convection-diffusion equation into Fick's Second Law in one dimension by replacing *z* with *u_max_t*, where *u_max_* is the velocity in the center of the channel. Nondimensionalizing this equation shows the mixing time is proportional to the characteristic length squared, in this case, $d_{sample}^{2}$

$$D\frac{\partial^{2}c(r,t)}{\partial r^{2}}=\frac{\partial c(r,t)}{\partial t}\to T_{mix}\propto d_{sample}^{2}/D.$$ (5)

**FIG. 13.**
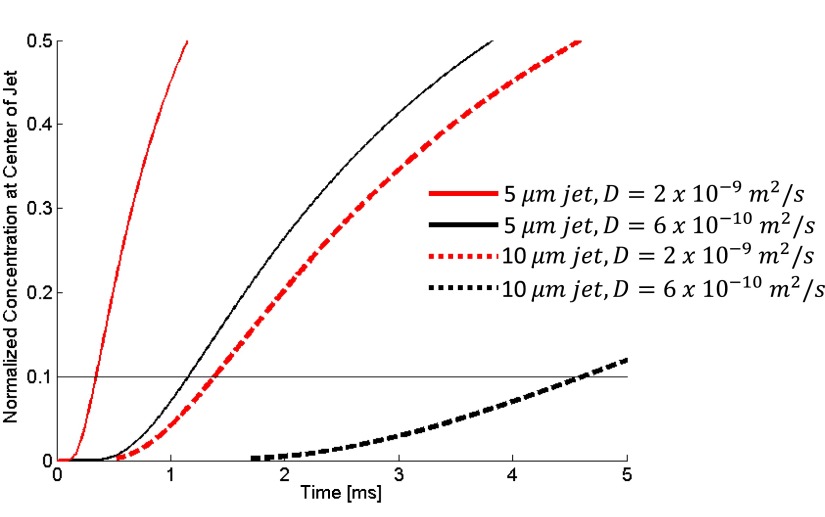
Simulation results of the concentration at the center divided by the initial reactant concentration for a 50 *μ*m channel with various sample jet diameters and diffusion coefficients. The coefficient of $D=2\times 10^{-9}m^{2}/s$ is similar to the diffusion coefficient for an ion such as iodide, while $D=6\times 10^{-10}m^{2}/s$ is close to the coefficient for glucose.

As shown in Figure 13 and Equation (3), mixing occurs significantly faster for smaller *d_sample_* The diffusion coefficient and threshold concentration of the reactant also influence the mixing time. For a given *d_sample_*, the mixing time can be reduced if a higher concentration of reactant is used in the sheath flow.

### D. Delay time

Another important characteristic of the mixing injector is the delay time, or the elapsed time after reaction initiation before the sample reaches the X-ray focus. Because this time determines which reaction intermediates are examined, it is necessary to understand how device design determines the delay time and enables its variation.

**FIG. 14.**
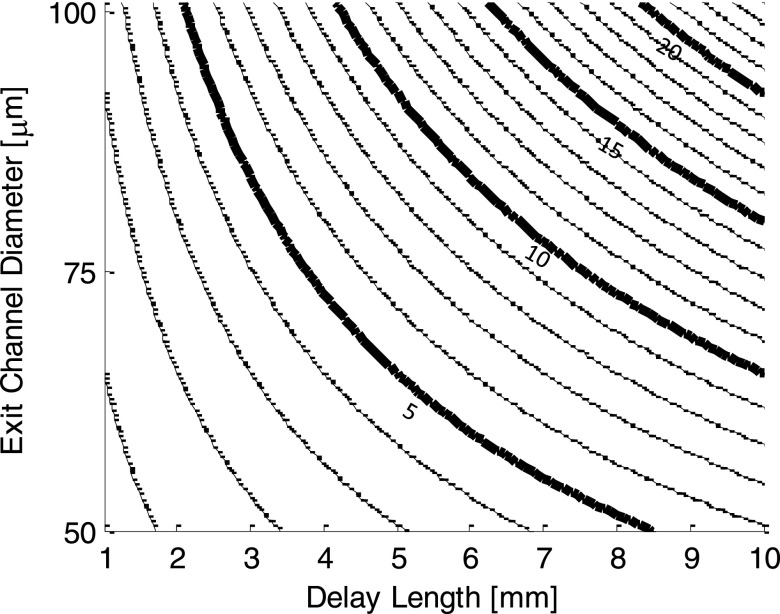
Delay time contours in ms as a function of the downstream inner capillary diameter (exit channel diameter) and delay length for 100 μ l/min total flow. The delay time in a completed device can be varied by approximately 10× by simply changing the flow rate. Plotted values can be scaled in this way by a factor of approximately 0.4–4.

The delay time experienced by the sample while inside the device is simply the time it takes to flow from the focusing region to the outlet of the capillary. This time depends only on the outlet channel length and overall sample flow rate, and is typically on the order of milliseconds. Upon exiting the capillary and entering the gas stream, the liquid jet is accelerated to approximately 10 m/s in less than 500 *μ*m while traveling through GDVN outlet and travels an additional several hundred microns to intersect the X-ray beam. This latter process introduces an additional delay of only 10 s of microseconds, which is insignificant compared to the delay times that occur inside the device, and can be neglected. With these approximations, the delay time is given by

$$t_{delay}=\frac{\pi L_{delay}{d_{channel}}^{2}}{8{\dot{V}}_{total}},$$ (6)

where *L_delay_* is the delay length (illustrated in Figure 12) and ${\dot{V}}_{total}$ is the total flow rate. Figure 14 shows a plot of delay time for various *D_Channel_* and *L_delay_*. By changing the total flow rate and fabricating devices with varying delay lengths, it is possible to achieve delay times from sub-millisecond to hundreds of milliseconds, enabling the study of reaction intermediates from processes that occur with a wide variety of different rates. Practical range for parameters that can be varied to affect the delay time are given in Table I.

**TABLE I.** Practical range of mixing injector parameters. The upper bound on the total flow rate is dependent on the beamline setup and is an estimate. Higher total flow rates may require more frequent cleaning of beamline sample catchers, costing experimental time.

Parameter		Units	Practical range
*L*	Delay length mm	mm	>1
*D*	Channel diameter	μm	20–100
${\dot{V}}_{total}$	Total flow rate	*μ*l/min	10–250*

### E. Hit rate

High hit rate is another desirable quality of a microfluidic mixer designed for XFEL experiments and is necessary if a high resolution structure is to be solved in a limited amount of experimental time. This is especially important in a mixing experiment when diffraction arises from a mixture of states, which may necessitate a higher number of indexed patterns.

A “hit” is a diffraction pattern with a minimum detectable number of Bragg peaks.^35^ For simplicity, we assume there is a hit any time a part of a crystal intersects the X-ray beam. This allows us to treat the crystals as point particles interacting with a larger effective beam, which has a diameter that is the sum of the beam diameter and the average crystal diameter. In this case, the hit rate is the product of the number density of diffracting crystals, ρ, and the effective irradiated volume, $V_{irr}^{*}$, which is the intersection of the liquid jet and the effective X-ray beam

$$Hit\;Rate=\rho V_{irr}^{*}.$$ (7)

**FIG. 15.**
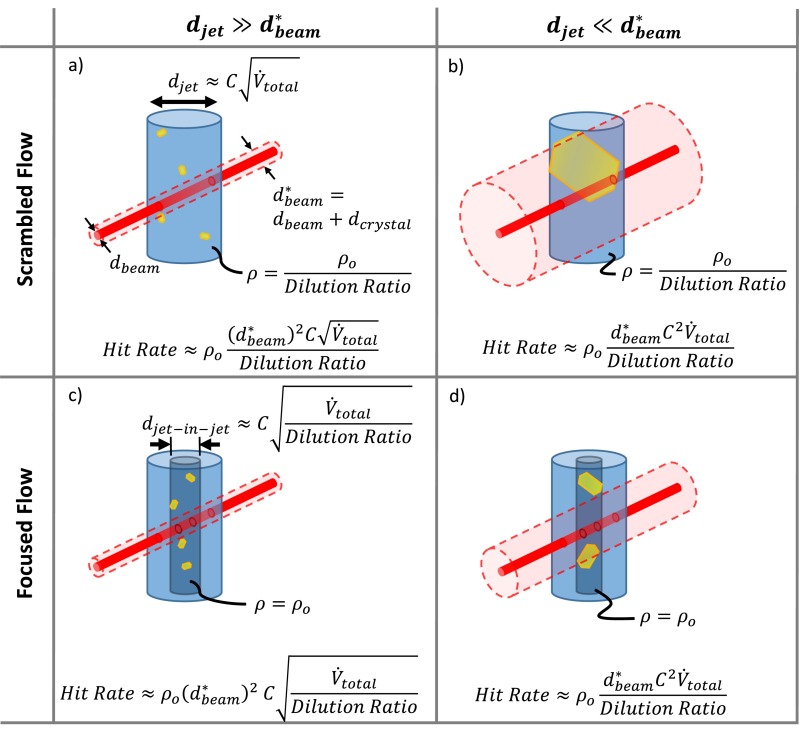
Illustration of the effective X-ray beam intersecting the free jet in four characteristic cases. (a) Scrambled flow where $d_{jet}\gg d_{beam}^{*}$. (b) Scrambled flow where $d_{jet}\ll d_{beam}^{*}$. (c) Focused flow where $d_{jet}\gg d_{beam}^{*}$. (d) Focused flow where $d_{jet}\ll d_{beam}^{*}$. The real X-ray beam is shown in solid red, while the effective beam is a transparent red cylinder. Crystals are in yellow, and the jet is blue. A darker blue cylinder represents the jet-within-jet in the flow focused regime.

For our devices, the free jet flow can fall into the two regimes observed in Figure 10, scrambling and flow focusing. These can be further examined in the limits where the jet diameter is much larger or smaller than the effective beam diameter. The jet diameter is given by

$$d_{jet}\approx C\sqrt{{\dot{V}}_{total}},$$ (8)

where C is constant for a given injector and helium flow rate.^16^ If the jet diameter is much larger than the beam diameter, not every crystal passes through $V_{irr}^{*}$. In the other limit, every crystal will pass through $V_{irr}^{*}$. These four characteristic cases are represented schematically in Figure 15. Since the maximum flow rate is limited by the beamline sample environment and is the same for any properly functioning injector, we express the hit rate for each case in terms of the total flow rate and the dilution ratio, where

$$Dilution\;Ratio=\frac{{\dot{V}}_{total}}{Sample\;Flow\;Rate}.$$ (9)

The hit rate equations in Figure 15 show how the hit rate scales for a given case depending on flow conditions. They are meant as a guide for experimenters who know their crystal size and hit rate using a standard GDVN, and would like to estimate their hit rate in a mixing experiment. An example of this type of calculation is given in the Appendix.

At first glance, it is tempting to compare the panels of Figure 15 to try to determine which case is ideal and design an experiment to operate in that mode. However, this is not a straightforward procedure. Clearly, to move from the $d_{jet}\gg d_{beam}^{*}$ limit to the $d_{jet}\ll d_{beam}^{*}$ limit, the beam size, crystal size, or flow rates must change, but some of these parameters are set by the experiment. Most frequently, the operating mode will be determined by the size of the crystals and the flow rates needed to achieve the desired mixing time.

In all cases, the hit rate is inversely related to the dilution ratio. Therefore, the constricted outlet of our device, which allows the same mixing time to be achieved with lower dilution, significantly increases the hit rate over what would be observed using the mixing injector described by Wang *et al.*^21^ This boosted hit rate allows more data to be collected in a given amount of experimental time and is essential for a successful XFEL mixing experiment.

### F. Considerations for balancing mixing time, hit rate, and sample dilution

High hit rates and short mixing times are features of an ideal mixing experiment. However, both of these parameters vary inversely with the dilution ratio

$$\mathrm{Hit Rat}e_{a),\;b),d)}\propto \frac{1}{\mathrm{Dilution Ratio}}\;\mathrm{or}\;\mathrm{Hit Rat}e_{c)}\propto \frac{1}{\sqrt{\mathrm{Dilution Ratio}}}$$

$$\tau_{\mathrm{mix}}\propto \frac{d_{\mathrm{channel}}^{2}}{\mathrm{Dilution Ratio}},$$

where a), b), c), and d) refer to the cases illustrated in Figure 15. Flowing a high dilution ratio produces fast mixing times but comes at the expense of hit rate. Given the high cost of and high competition for experimental time at an XFEL, any time-resolved SFX experimenter must choose flow parameters to carefully balance the desired time resolution and hit rate.

## V. CONCLUSION

We fabricated and characterized a microfluidic mixing injector for XFEL experiments that meets the demands of the XFEL beamline environment while efficiently and repeatedly mixing the sample. A manuscript describing the first use of this new mixing injector at CXI/LCLS is in preparation.^36^ The concentrically bonded glass capillaries are large enough, 50 or 75 *μ*m, to resist clogging by clusters of crystals. They withstand high pressures and a wide range of pH and salt concentrations to form a robust, dependable device. We observed submillisecond mixing times in fluorescence experiments. While particles can either be focused or scrambled in the free jet, the reduced-diameter outlet of this device ensures a high hit rate either way, representing a significant improvement over previous work. With its short premixing times, observable reaction times from submillisecond to hundreds of milliseconds, and low dilution ratios, this mixing injector is a substantial step forward in the field of time-resolved crystallography. XFEL experiments with this device will capture never-before-seen intermediate states from chemically activated reactions and open up a wealth of new knowledge of protein function.

## APPENDIX: EXAMPLE HIT RATES IN MIXING INJECTOR

To demonstrate the application of the hit rate equations given in Figure 15 for estimating the hit rate, we apply them to two different example crystal systems: one with $d_{jet}\gg d_{beam}^{*}$ and one with $d_{jet}\ll d_{beam}^{*}$.

First, we examine the case of $d_{jet}\gg d_{beam}^{*}$, which would occur for very small crystals. For example, consider a sample in this regime with a hit rate of 10% when flowed at 10 *μ*l/min through a standard GDVN. If this sample instead flows through our mixing injector with a 30:1 dilution ratio, total flow rate of 150 *μ*l/min, and scrambled flow as in Figure 15(a), the hit rate would be 1.3%. If the mixer operated in flow focusing mode, as in Figure 15(c), the hit rate would be 7%.

Now consider a *different* sample in the regime where $d_{jet}\ll d_{beam}^{*}$, which could occur for crystals larger than several microns or for larger beams. Imagine that this sample also has a 10% hit rate in a GDVN at 10 *μ*l/min. If this sample flowed through our mixing injector with a 30:1 dilution ratio, a total flow rate of 150 *μ*l/min, a scrambled flow as in Figure 15(b), the hit rate would be 5%. With this simplified analysis, no additional benefit is gained for this system by flow focusing in the $d_{jet}\ll d_{beam}^{*}$ regime. However, in reality, flow focusing will result in more direct hits with stronger diffraction.

## References

[1] J. R. Helliwell , “ Synchrotron X-radiation protein crystallography: instrumentation, methods and applications,” Rep. Prog. Phys. , 1403–1497 (1984). 10.1088/0034-4885/47/11/001

[2] K. Wüthrich , “ Protein structure determination in solution by NMR spectroscopy,” J. Biol. Chem. , 22059–22062 (1990). 2266107

[3] F. Schotte , M. Lim , T. A. Jackson , A. V. Smirnov , J. Soman , J. S. Olson , G. N. Phillips, Jr. , M. Wulff , and P. A. Anfinrud , “ Watching a protein as it functions with 150-ps time-resolved x-ray crystallography,” Science , 1944–1947 (2003). 10.1126/science.1078797 12817148

[4] K. A. Henzler-Wildman , M. Lei , V. Thai , S. J. Kerns , M. Karplus , and D. Kern , “ A hierarchy of timescales in protein dynamics is linked to enzyme catalysis,” Nature , 913–916 (2007). 10.1038/nature06407 18026087

[5] S. J. Benkovic and S. Hammes-Schiffer , “ A perspective on enzyme catalysis,” Science , 1196–1202 (2003). 10.1126/science.1085515 12947189

[6] U. K. Genick , G. E. O. Borgstahl , K. Ng , Z. Ren , C. Pradervand , P. M. Burke , V. Srajer , T. Teng , W. Schildkamp , D. E. McRee , K. Moffat , and E. D. Getzoff , “ Structure of a protein photocycle intermediate by millisecond time—Resolved crystallography,” Science , 1471–1475 (1997). 10.1126/science.275.5305.1471 9045611

[7] J. Hajdu and I. Andersson , “ Fast crystallography and time-resolved structures,” Annu. Rev. Biophys. Biomol. Struct. , 467–498 (1993). 10.1146/annurev.bb.22.060193.002343 8347998

[8] S. Geremia , M. Campagnolo , N. Demitri , and L. N. Johnson , “ Simulation of diffusion time of small molecules in protein crystals,” Structure , 393–400 (2006). 10.1016/j.str.2005.12.007 16531224

[9] S. Boutet , L. Lomb , G. J. Williams , T. R. Barends , A. Aquila , R. B. Doak , U. Weierstall , D. P. DePonte , J. Steinbrener , R. L. Shoeman , M. Messerschmidt , A. Barty , T. A. White , S. Kassemeyer , R. A. Kirian , M. M. Seibert , P. A. Montanez , C. Kenney , R. Herbst , P. Hart , J. Pines , G. Haller , S. M. Gruner , H. T. Philipp , M. W. Tate , M. Hromalik , L. J. Koerner , N. van Bakel , J. Morse , W. Ghonsalves , D. Arnlund , M. J. Bogan , C. Caleman , R. Fromme , C. Y. Hampton , M. S. Hunter , L. C. Johansson , G. Katona , C. Kupitz , M. Liang , A. V. Martin , K. Nass , L. Redecke , F. Stellato , N. Timneanu , D. Wang , N. A. Zatsepin , D. Schafer , J. Defever , R. Neutze , P. Fromme , J. C. Spence , H. N. Chapman , and I. Schlichting , “ High-resolution protein structure determination by serial femtosecond crystallography,” Science , 362–364 (2012). 10.1126/science.1217737 22653729 PMC3788707

[10] I. Schlichting , “ Serial femtosecond crystallography: The first five years,” IUCrJ , 246–255 (2015). 10.1107/S205225251402702X PMC4392417 25866661

[11] D. P. DePonte , U. Weierstall , K. Schmidt , J. Warner , D. Starodub , J. C. H. Spence , and R. B. Doak , “ Gas dynamic virtual nozzle for generation of microscopic droplet streams,” J. Phys. D. Appl. Phys. , 195505 (2008). 10.1088/0022-3727/41/19/195505

[12] U. Weierstall , D. James , C. Wang , T. A. White , D. Wang , W. Liu , J. C. H. Spence , R. Bruce Doak , G. Nelson , P. Fromme , R. Fromme , I. Grotjohann , C. Kupitz , N. A. Zatsepin , H. Liu , S. Basu , D. Wacker , G. W. Han , V. Katritch , S. Boutet , M. Messerschmidt , G. J. Williams , J. E. Koglin , M. Marvin Seibert , M. Klinker , C. Gati , R. L. Shoeman , A. Barty , H. N. Chapman , R. A. Kirian , K. R. Beyerlein , R. C. Stevens , D. Li , S. T. A. Shah , N. Howe , M. Caffrey , and V. Cherezov , “ Lipidic cubic phase injector facilitates membrane protein serial femtosecond crystallography,” Nat. Commun. , 3309 (2014). 10.1038/ncomms4309 24525480 PMC4061911

[13] C. G. Roessler , R. Agarwal , M. Allaire , R. Alonso-Mori , B. Andi , J. F. R. Bachega , M. Bommer , A. S. Brewster , M. C. Browne , R. Chatterjee , E. Cho , A. E. Cohen , M. Cowan , S. Datwani , V. L. Davidson , J. Defever , B. Eaton , R. Ellson , Y. Feng , L. P. Ghislain , J. M. Glownia , G. Han , J. Hattne , J. Hellmich , A. Heroux , M. Ibrahim , J. Kern , A. Kuczewski , H. T. Lemke , P. Liu , L. Majlof , W. M. McClintock , S. Myers , S. Nelsen , J. Olechno , A. M. Orville , N. K. Sauter , A. S. Soares , S. M. Soltis , H. Song , R. G. Stearns , R. Tran , Y. Tsai , M. Uervirojnangkoorn , C. M. Wilmot , V. Yachandra , J. Yano , E. T. Yukl , D. Zhu , and A. Zouni , “ Acoustic injectors for drop-on-demand serial femtosecond crystallography,” Structure , 631–640 (2016). 10.1016/j.str.2016.02.007 26996959 PMC4920001

[14] S. Oghbaey , A. Sarracini , H. M. Ginn , O. Pare-Labrosse , A. Kuo , A. Marx , S. W. Epp , D. A. Sherrell , B. T. Eger , Y. Zhong , R. Loch , V. Mariani , R. Alonso-Mori , S. Nelson , H. T. Lemke , R. L. Owen , A. R. Pearson , D. I. Stuart , O. P. Ernst , H. M. Mueller-Werkmeister , and R. J. D. Miller , “ Fixed target combined with spectral mapping: Approaching 100% hit rates for serial crystallography,” Acta Cryst. D , 944–955 (2016). 10.1107/S2059798316010834 PMC5937680 27487825

[15] U. Weierstall , “ Liquid sample delivery techniques for serial femtosecond crystallography,” Phil. Trans. R. Soc. B , 20130337 (2014). 10.1098/rstb.2013.0337 24914163 PMC4052872

[16] A. Gañán-Calvo , “ Generation of steady liquid microthreads and micron-sized monodisperse sprays in gas streams,” Phys. Rev. Lett. , 285–288 (1998). 10.1103/PhysRevLett.80.285

[17] M. A. Herrada , A. M. Gañán-Calvo , A. Ojeda-Monge , B. Bluth , and P. Riesco-Chueca , “ Liquid flow focused by a gas: Jetting, dripping, and recirculation,” Phys. Rev. E - Stat. Nonlinear Soft Matter Phys. , 036323 (2008). 10.1103/PhysRevE.78.036323 18851159

[18] C. Kupitz , S. Basu , I. Grotjohann , R. Fromme , N. A. Zatsepin , K. N. Rendek , M. S. Hunter , R. L. Shoeman , T. A. White , D. Wang , D. James , J.-H. Yang , D. E. Cobb , B. Reeder , R. G. Sierra , H. Liu , A. Barty , A. L. Aquila , D. Deponte , R. A. Kirian , S. Bari , J. J. Bergkamp , K. R. Beyerlein , M. J. Bogan , C. Caleman , T.-C. Chao , C. E. Conrad , K. M. Davis , H. Fleckenstein , L. Galli , S. P. Hau-Riege , S. Kassemeyer , H. Laksmono , M. Liang , L. Lomb , S. Marchesini , A. V. Martin , M. Messerschmidt , D. Milathianaki , K. Nass , A. Ros , S. Roy-Chowdhury , K. Schmidt , M. Seibert , J. Steinbrener , F. Stellato , L. Yan , C. Yoon , T. A. Moore , A. L. Moore , Y. Pushkar , G. J. Williams , S. Boutet , R. B. Doak , U. Weierstall , M. Frank , H. N. Chapman , J. C. H. Spence , and P. Fromme , “ Serial time-resolved crystallography of photosystem II using a femtosecond X-ray laser,” Nature , 261–265 (2014). 10.1038/nature13453 25043005 PMC4821544

[19] J. Tenboer , S. Basu , N. Zatesepin , K. Pande , D. Milathianaka , M. Frank , M. Hunter , S. Boutet , G. J. Williams , J. E. Koglin , D. Oberthuer , M. Heymann , C. Kupitz , C. Conrad , J. Coe , S. Roy-Chowdhury , U. Weierstall , D. James , D. Wang , T. Grant , A. Barty , O. Yefanov , J. Scales , C. Gati , C. Seuring , V. Srajer , R. Henning , P. Schwander , R. Fromme , A. Ourmazd , K. Moffat , J. J. Van Thor , J. C. H. Spence , P. Fromme , H. N. Chapman , and M. Schmidt , “ Time-resolved serial crystallography captures high-resolution intermediates of photoactive yellow protein,” Science , 1242–1246 (2014). 10.1126/science.1259357 25477465 PMC4361027

[20] M. Schmidt , “ Mix and inject: Reaction initiation by diffusion for time-resolved macromolecular crystallography,” Adv. Condens. Matter Phys. , 1–10 (2013). 10.1155/2013/167276

[21] D. Wang , U. Weierstall , L. Pollack , and J. C. H. Spence , “ Liquid mixing jet for XFEL study of chemical kinetics,” J. Synchrotron Radiat. , 1364–1366 (2014). 10.1107/S160057751401858X 25343806 PMC4211133

[22] J. Knight , A. Vishwanath , J. Brody , and R. Austin , “ Hydrodynamic focusing on a silicon chip: Mixing nanoliters in microseconds,” Phys. Rev. Lett. , 3863–3866 (1998). 10.1103/PhysRevLett.80.3863

[23] H. Y. Park , X. Qiu , E. Rhoades , J. Korlach , L. W. Kwok , W. R. Zipfel , W. W. Webb , and L. Pollack , “ Achieving uniform mixing in a microfluidic device: hydrodynamic focusing prior to mixing,” Anal. Chem. , 4465–4473 (2006). 10.1021/ac060572n 16808455

[24] S. Boutet and G. J. Williams , “ The coherent x-ray imaging (CXI) instrument at the linac coherent light source (LCLS),” New J. Phys. , 035024 (2010). 10.1088/1367-2630/12/3/035024

[25] K. Tono , E. Nango , M. Sugahara , C. Song , J. Park , T. Tanaka , R. Tanaka , Y. Joti , T. Kameshima , S. Ono , T. Hatsui , E. Mizohata , M. Suzuki , T. Shimamura , Y. Tanaka , S. Iwata , and M. Yabashi , “ Diverse application platform for hard X-ray diffraction in SACLA (DAPHNIS): Application to serial protein crystallography using an X-ray free-electron laser,” J. Synchrotron Radiat. , 532–537 (2015). 10.1107/S1600577515004464 25931065 PMC4817517

[26] N. L. Rosidi , J. Zhou , S. Pattanaik , P. Wang , W. Jin , M. Brophy , W. L. Olbricht , N. Nishimura , and C. B. Schaffer , “ Cortical microhemorrhages cause local inflammation but do not trigger widespread dendrite degeneration,” PLoS One , e26612 (2011). 10.1371/journal.pone.0026612 22028924 PMC3197572

[27] D. James , “Injection methods and instrumentation for serial x-ray free electron laser experiments,” Ph.D. dissertation, Arizona State University (2015).

[28] Q. S. Hanley , V. Subramaniam , D. J. Arndt-Jovin , and T. M. Jovin , “ Fluorescence lifetime imaging: Multi-point calibration, minimum resolvable differences, and artifact suppression,” Cytometry , 248–260 (2001). 10.1002/1097-0320(20010401)43:4<248::AID-CYTO1057>3.0.CO;2-Y 11260592

[29] K. R. Beyerlein , L. Adriano , M. Heymann , R. Kirian , J. Knoška , F. Wilde , H. N. Chapman , and S. Bajt , “ Ceramic micro-injection molded nozzles for serial femtosecond crystallography sample delivery,” Rev. Sci. Instrum. , 125104 (2015). 10.1063/1.4936843 26724070

[30] N. Rebollo-Muñoz , A. J. Acero , J. Z. Marcos de León , J. M. Montanero , and A. M. Gañán-Calvo , “ A hybrid flow focusing nozzle design to produce micron and sub-micron capillary jets,” Int. J. Mass Spectrom. , 32–48 (2016). 10.1016/j.ijms.2016.03.005

[31] M. Schmidt , S. Rajagopal , Z. Ren , and K. Moffat , “ Application of singular value decomposition to the analysis of time-resolved macromolecular x-ray data,” Biophys. J. , 2112–2129 (2003). 10.1016/S0006-3495(03)75018-8 12609912 PMC1302779

[32] B. W. Matthews , “ Solvent content of protein crystals,” J. Mol. Biol. , 491–497 (1968). 10.1016/0022-2836(68)90205-2 5700707

[33] M. M. Tomadakis and S. V. Sotirchos , “ Transport properties of random arrays of freely overlapping cylinders with various orientation distributions,” J. Chem. Phys. , 616 (1993). 10.1063/1.464604

[34] S. A. Pabit and S. J. Hagen , “ Laminar-flow fluid mixer for fast fluorescence kinetics studies,” Biophys. J. , 2872–2878 (2002). 10.1016/S0006-3495(02)75296-X 12414719 PMC1302371

[35] A. Barty , R. A. Kirian , F. R. N. C. Maia , M. Hantke , C. H. Yoon , T. A. White , and H. Chapman , “ Cheetah: Software for high-throughput reduction and analysis of serial femtosecond X-ray diffraction data,” J. Appl. Crystallogr. , 1118–1131 (2014). 10.1107/S1600576714007626 24904246 PMC4038800

[36] G. D. Calvey *et al.*, “Functional motions of Beta-lactamase revealed at XFEL using time-resolved WAXS,” (unpublished).

